# Spatial diversity of atmospheric moisture transport and climate teleconnections over Indian subcontinent at different timescales

**DOI:** 10.1038/s41598-024-62760-2

**Published:** 2024-05-31

**Authors:** Akash Singh Raghuvanshi, Ankit Agarwal

**Affiliations:** Department of Hydrology, Indian Institute of Technology, Roorkee, 247667 India

**Keywords:** Atmospheric moisture transport, Wavelet coherence, Global wavelet coherence, Teleconnections, Integrated water vapor transport, India, Climate sciences, Hydrology

## Abstract

Regional weather and climate are generally impacted by global climatic phenomenon′s. Understanding the impact of global climate phenomenon′s on an atmospheric branch of the hydrological cycle is crucial to make advances in skillful precipitation forecast. The present study adopts a multiscale approach based on wavelets for unravelling the linkages between teleconnections and atmospheric moisture transport over homogeneous regions of Indian sub-continent. We investigated linkages between atmospheric moisture transport quantified as monthly integrated water vapor transport (IVT) during 1951–2022 over selected homogeneous regions and eight large scale climate oscillations using wavelet and global wavelet coherence. Our results indicate significant heterogeneity in linkages across different regions and across multiple timescales. In particular, the Indian Ocean Dipole (IOD) influence monthly IVT at intra-annual to inter-annual scale over all regions. The El Niño–Southern Oscillation (ENSO) have strong connection to monthly IVT at inter-annual scale whereas over west central region both IOD and ENSO strongly influence IVT at inter-decadal scale. While the Atlantic Multi-Decadal Oscillation and Pacific Decadal Oscillation have an impact on IVT in the north-east and southern regions, the Arctic Oscillation and North Atlantic oscillation have a strong inter-annual connection to IVT, majorly in the northwest and hilly regions. Overall, the methodology offers an effective approach for capturing the dynamics of atmospheric moisture transport in time–frequency space and provide a practical reference for prediction of atmospheric moisture transport linked precipitation over different regions of Indian subcontinent.

## Introduction

Atmospheric moisture transport is the principal component of atmospheric branch of hydrological cycle and plays a significant role in the global climate system. Several research in past^1–3^ have examined the potential impact of atmospheric moisture transport across the regions at both local and global scales. Atmospheric moisture transport links precipitation over the continents to evaporation from the land and oceans^4,5^. In addition, Mahoney et al. and Viale et al.^6,7^ demonstrate that atmospheric moisture transport has a substantial influence in altering the intensity and frequency of precipitation. Over Indian subcontinent, seasonal variability in column integrated water vapour can explain the seasonal variability of precipitation^8,9^. Additionally, powerful low-level winds from incoming and outgoing moisture flux regulate net moisture convergence over India^10^, which further influences regional variability of precipitation over India. Furthermore, a strong correlation between atmospheric moisture transport and precipitation has been observed across India^11–13^. Numerous indices have been developed for quantifying atmospheric moisture transport. Due to the accessibility of water vapor images from satellites, many studies have previously employed column integrated water vapor (IWV) data from microwave sensors^14–16^. The column integrated water vapour transport (IVT), which incorporates horizontal winds in its computation, is now the standard metric to quantify moisture transport because it was later demonstrated that IWV does not account for the flux component, i.e., wind. The ability to more precisely detect better associations with precipitation and are more adeptly anticipated by numerical weather prediction models are two further benefits of IVT over IWV^17–21^.

In general, large-scale teleconnection patterns are related to exceptional tropical or subtropical heat sources and are linked to climatic variability at different spatiotemporal scales^22^. Numerous indices are developed to identify such large-scale teleconnection patterns^23,24^. Furthermore, it has been accepted that variability in the large-scale atmospheric patterns, particularly moisture transport and convergence, may have an impact on where and how much precipitation occurs^25,26^. According to Rogers et al.^27^ large scale climate patterns strongly modulate atmospheric moisture transport at interannual to multidecadal scales. In recent years, efforts have been made to quantify the relationships between atmospheric moisture transport and large-scale climatic indices. Although, most studies have confined themselves to investigating the effects of few teleconnection patterns on atmospheric moisture transport. For instance, the role of atmospheric moisture transport during the ENSO was studied by Baier et al. and Castillo et al.^28,29^. The effects of the Arctic (AO), Antarctic (AAO), Pacific-North American (PNA) and ENSO on moisture transport during the peak precipitation months were examined by Vázquez et al.^30^. Nieto et al.^31^ looked into how moisture transport was impacted by AAO and AO patterns. The impact of the North Atlantic oscillation (NAO), the AO, and the North Pacific oscillation (NPO) on the atmospheric moisture budget was examined by Rogers et al.^27^.

Over Indian subcontinent, the variability in precipitation at different time scales are linked to several oscillations, including AO, Atlantic Multi-Decadal Oscillation (AMO), NAO, Pacific Decadal Oscillation (PDO), ENSO, IOD etc^32–37^. For instance, winter precipitation dipole pattern over and central India and western Himalaya is dynamically linked to positive phase of the AO^38^ which is characterized by the quasi-barotropic Euro-Atlantic and Siberian High. Since the positive phase of AO is linked to the Rossby wave with wavenumber 2 and are accompanied by wave train pattern across Europe. These wave train patterns strengthened the jet stream over middle east and initiates the moisture transport by producing an anticyclonic circulation over Arabian sea^38^. AMO is linked to Indian summer monsoon (ISM) at multi-decadal time scale^35^. The AMO causes a persistent strengthening of the meridional gradient of TT^39^ by creating a positive tropospheric temperature (TT) anomaly over Eurasia during the northern late summer/autumn. This results in a late summer monsoon withdrawal and a persistent increase in seasonal monsoonrainfall^35^. A similar physical mechanism was also crucial in linking NAO and Indian summer precipitation on an inter-annual time scale^35^. On decadal to multidecadal timescales, PDO is linked to variability in precipitation over India^40–42^. Several physical mechanisms have been proposed in the past to link PDO to precipitation over the Indian subcontinent. For example, using the Walker and Hadley circulations, Krishnamurthy and Krishnamurthy^42^ proposed a relationship between the PDO and ISM, with the tropical Pacific acting as a bridge between the North Pacific and the Indian monsoon. On the contrary, study by Krishnamurthy and Krishnamurthy^40^ found that ISM has different relationships with the traditional PDO and the decadal component of the PDO. The warm phase of the PDO's pure decadal variability is linked to a drought in India's west central region. The traditional warm PDO index, on the other hand, is associated with low rainfall across most of India. The warm phase of the pure decadal PDO restricts moisture flow beyond 20°N over the ISM region via North Pacific meridional winds, resulting in less rainfall over west central India^40^.

ENSO and IOD are linked to interannual variability in precipitation over India^43^. In the case of ENSO, El Nino (La Nina) conditions over the Pacific Ocean are frequently associated with weak (strong) ISM. The entire Walker circulation shifts eastward during El Nino events, with the falling branch of the Walker cell on the western Indian Ocean shifting eastward over the Indian subcontinent, inhibiting convection^44,45^. During La Nina years, the entire Walker circulation shifts slightly westward, which aids in increasing convection over the Indian subcontinent. During La Nina years, the entire Walker circulation shifts slightly westward, which aids in enhancing convection over the Indian subcontinent. Many other studies^46,47^ claimed that El Nino conditions do not suppress ISMR directly via the falling branch of the Walker circulation, but rather that variations in the Walker circulation strengthen the meridional Hadley circulation descent over the Indian subcontinent. As a result, ENSO may have an impact on ISM through interactions between the Walker and Hadley circulations. Positive IOD events influence the region's meridional circulation through exceptional convergence patterns over the Bay of Bengal, strengthening the monsoon with exceptional positive rainfall over the Indian subcontinent, whereas negative IOD events weaken rainfall^48^.

To the author's knowledge, majority of previous studies in the Indian subcontinent were focussed on regional variability of hydroclimatic variables (mainly precipitation) and its connection with climate oscillations^34,37, 39, 49–55^. However, it is still unclear how large-scale teleconnections affects atmospheric moisture transport across different geographical regions of India. Furthermore, it is essential to comprehend the relationships between climatic indices and moisture transport over a range of timescales in order to improve the predictability of atmospheric moisture transport and associated precipitation across the Indian subcontinent region. The present study, which is the first of its kind, unravels the connections between climate teleconnection indices and atmospheric moisture transport by providing an in-depth analysis of the timescales at which the atmospheric moisture transport across the Indian subcontinent region is influenced by large-scale climate oscillations.

This paper is structured as follows: "Study area and data" section covers the study region and data used, "Methods and methodology" section details the methodology employed, "Results and discussion" section displays the results and provides a brief discussion, and "Conclusions" section provides a summary of the key finding and conclusions.

## Study area and data

**Figure 1:**
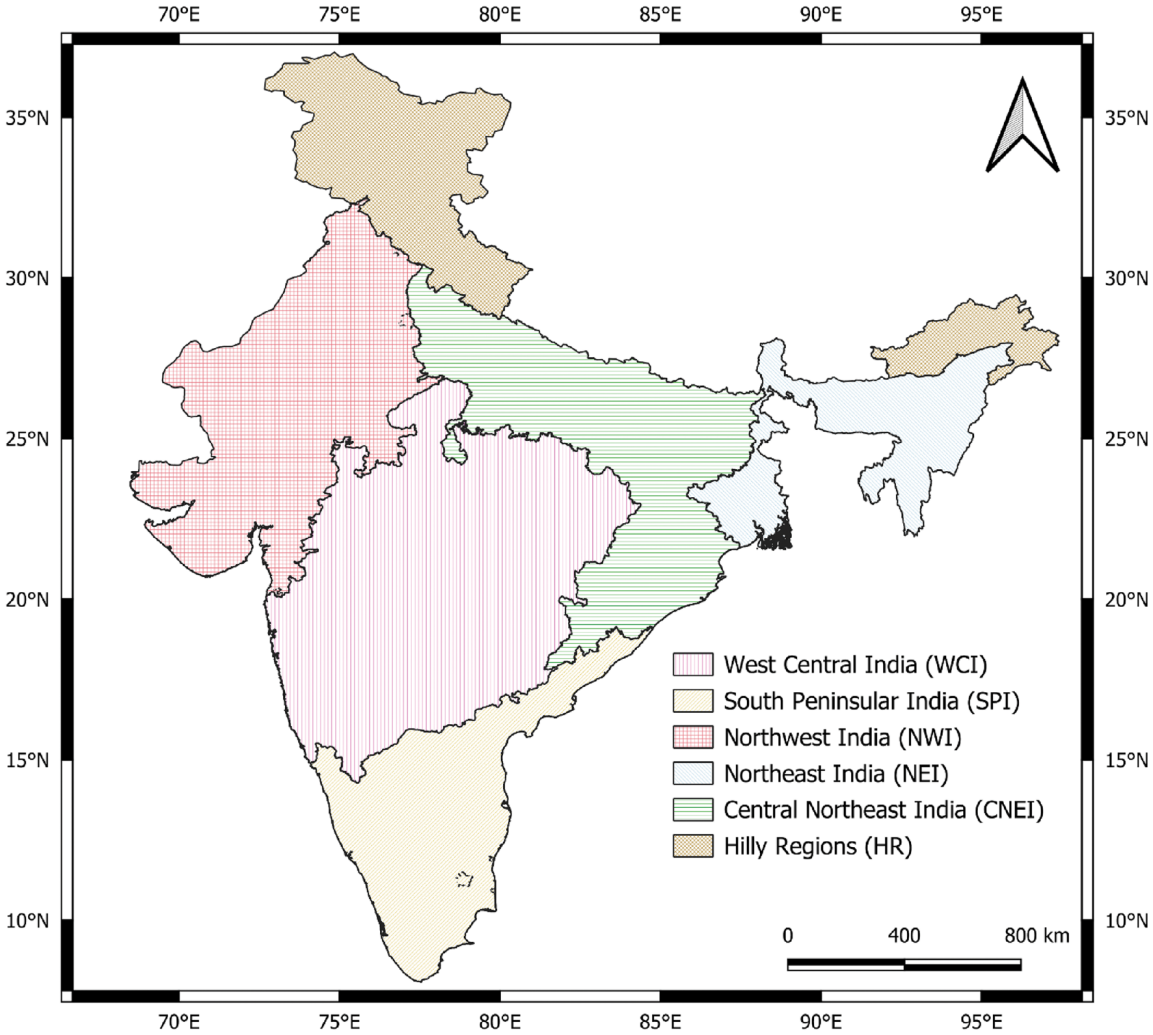
Map for meteorologically homogeneous regions of India selected for examining linkages. QGIS 3.30.0 software is used to create the map (https://www.qgis.org/en/site/).

India is a vast country that exhibits significant variation in climate characteristics. Based on geographical extent, Indian sub-continent is around 3.287 million km^2^. The Indian subcontinent is categorized into five homogeneous zones based on similarities in precipitation characteristics, according to the India Meteorological Department (IMD) and the Indian Institute of Tropical Meteorology (IITM) research study RR-138^56^. West Central India (WCI), South Peninsular India (SPI), Northwest India (NWI), Central Northeast India (CNEI), Northeast India (NEI), and the hilly areas (HR), which encompass the Himalayas in the north and north-east, are the six homogeneous regions depicted in Fig. 1.

In the present work, we used recently developed European Centre for Medium-Range Weather Forecasts (ECMWF) version 5 Reanalysis (ERA5) data^57^, an extension to the 50s^58^ for 1951–2022 due to its high spatiotemporal resolution on a global scale. Compared to other reanalysis products, recent studies^59–62^ have determined ERA-5 to be suitable for hydrological and meteorological assessments in the Indian subcontinent. The latitude–longitude grid of the ERA5 has a spatial resolution of 0.25° × 0.25°. To measure the amount of moisture transported, specific humidity and horizontal wind fields (zonal and meridional wind speed) at various vertical tropospheric levels (1000-300 hPa; 20 total pressure levels) are retrieved at 6-hourly temporal resolution. A 0.25° × 0.25° latitude–longitude grid of daily precipitation data developed by the India Meteorological Department (IMD) for the years 1951 to 2022 was also utilized. Pai et al.^63^ provided the specifics on how the precipitation data was prepared.

**Figure 2:**
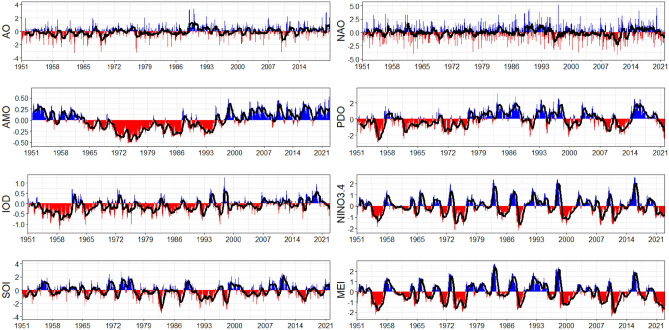
The positive and negative monthly values (blue and red, respectively) of the chosen teleconnection indices from 1951 to 2022. The climatic oscillation's 10-month moving average is depicted by a thick, black line.

Furthermore, we employ time series of several climate indices accessible at monthly temporal scale for the period 1951–2022, to explore the links between climatic teleconnections and atmospheric moisture transport. Arctic Oscillation (AO) signifies the direction and intensity of westerly winds in the Arctic region. Figure 2 highlights variability in AO (positive and negative phases) at both shorter (monthly scale) and longer (year to decade) time scales. Information about the Arctic Oscillation (AO), also referred to as the Northern Hemisphere annular mode, is available at https://www.ncei.noaa.gov/access/monitoring/ao/. The Atlantic Multi-Decadal Oscillation (AMO), which can be found at https://climatedataguide.ucar.edu/climate-data/atlantic-multi-decadal-oscillation-amo, is recognized as a coherent phase of natural variability occurring in the North Atlantic Ocean. Figure 2 depicts the variability of AMO over a multidecadal time span. As a result, the warm and cool AMO phases span approximately 20–30 years (Fig. 2). Monthly sea surface temperature (SST) anomaly data across the 17° E–120^o^ W, 5^o^ S–5^o^ N region are used to calculate the NINO3.4 index, which is frequently used to identify ENSO events. NINO3.4 index data is available at https://psl.noaa.gov/gcos_wgsp/Timeseries/Data/nino34.long.anom.data. The surface air pressure differential between Tahiti and Darwin is used to calculate the Southern Oscillation index (SOI), which also provides an indication of ENSO intensity and its development. This information and relevant data can be found at https://crudata.uea.ac.uk/cru/data/soi/soi.dat. Compared to earlier ENSO indices, the Multivariate ENSO index (MEI), which is based on numerous variables that can be seen over the tropical Pacific, provides more information about ENSO phenomenon and can be accessed through https://psl.noaa.gov/enso/mei/. In the past, there have been times when warmer phase of ENSO (El Nino; indicated by blue in Fig. 2) are followed by cooler phase of ENSO (La Nina; indicated by red in Fig. 2). Further, it can be observed that these phases usually occur in every 2–7 years (Fig. 2). The Dipole Mode Index (DMI), which is defined as the difference between the tropical western and tropical south-eastern Indian Ocean surface temperature anomaly, is used to measure the Indian Ocean Dipole (IOD), which is caused by the tropical Indian Ocean's dipole mode. The IOD is a coupled ocean–atmosphere phenomenon that occurs in the equatorial Indian Ocean, similar to ENSO. The IOD is thought to be linked to ENSO events via a westward extension of the Walker Circulation and associated Indonesian throughflow (the flow of warm tropical ocean water from the Pacific into the Indian Ocean). As a result, positive IOD events are frequently associated with El Nino, and negative IOD events with La Nina (depicted in Fig. 2). More information regarding the DMI data can be obtained from https://psl.noaa.gov/gcos_wgsp/Timeseries/Data/dmi.had.long.data. The distinction between a subtropical high and a subpolar low in terms of sea pressure is known as the North Atlantic Oscillation (NAO) index. NAO is one of the primary modes of variation, particularly during the winter in the Northern Hemisphere. Similar to AO, NAO also exhibit variation at shorter (monthly) and longer (year to decade) time scales (Fig. 2). Studies in past has supported this close association between NAO and AO^34,64^. NAO dataset is accessible from https://www.ncei.noaa.gov/access/monitoring/nao/. The Pacific Decadal Oscillation (PDO) is a long-lasting pattern of climate variation in the Pacific that mimics El-Nino. Figure 2 depicts variability in PDO primarily at the decadal to multidecadal time scale. Further information about the PDO data is available at https://www.ncei.noaa.gov/access/monitoring/pdo/.

## Methods and methodology

Numerous techniques, including correlation^65^, regression analysis^66^, empirical orthogonal functions^67^ and principal component analysis^68^, have been used over the years to investigate the relationships between climatic patterns and atmospheric variables. However, all of these statistical techniques, fall short in their ability to capture the multi-scale interaction and feedback between long-range climate patterns and atmospheric variables^52^. This understanding is crucial because energy in climatic systems is transported and stored in diverse ways over a range of temporal scales as a result of interactions between interrelated sub-components at various scales^69,70^. As a result, multiscale feedbacks and interactions have garnered a lot of attention in the study of climate dynamics^70,71^. In recent decades, the wavelet coherence approach has been widely used in the field of hydrometeorology and has come to be recognized as one of the effective and useful methods for processing signals and time series. Moreover, wavelet coherence has become an emerging technique for examining how climate patterns affect hydro-meteorological variables at various temporal scales. For instance, Das et al.^34^ employed the wavelet coherence approach to examine the connection between large scale climatic patterns and precipitation in India. The study reported IOD and ENSO being prominent at inter-annual scale. Coherently, Kurths et al.^52^ analysed teleconnection influence on Indian precipitation using similar multiscale approach. Further, similar wavelet coherence methodology has been reported across the globe^72–75^.

### Integrated water vapor transport (IVT)

The IVT is estimated in the Eulerian framework^76^ by integrating zonal (u), meridional (v) wind speeds in m/s, and specific humidity q in kg/kg across vertical pressure levels (1000–300 hPa) using following equation:

1 $$IVT = \sqrt {IVT_{x}^{2} + IVT_{y}^{2} } = \sqrt {\left( {\frac{1}{g}\mathop \smallint \limits_{1000}^{300} qu dp} \right)^{2} + \left( {\frac{1}{g}\mathop \smallint \limits_{1000}^{300} qv dp} \right)^{2} }$$

where dp is the pressure difference (in Pa) and g is the acceleration caused by gravity (9.81 m/s^2^).

### Wavelet analysis

Any continuous geophysical variable's temporal data series is essentially a superposition of fluctuations that occur at multiple scales^52^. These patterns are the result of several physical processes, and by partitioning the variability at various scales, it is possible to identify and describe the underlying processes^77,78^. The interactions timing between physical drivers and fluxes has been successfully characterized by wavelets^79^. When a signal is wavelet transformed, it is divided into several components with predetermined spectral bandwidths and core frequencies^52^. The wavelet transform was developed using a theory that is quite similar to the Fourier transformations. Nevertheless, it offers far greater versatility in examining all of the frequencies contained in a time series^80^. In the fields of hydro-climatology and signal processing, this approach has been widely used^81–83^. Wavelet transformations (WT) generally come in two flavours: continuous WT (CWT) and discrete WT (DWT). Since the scaling factor is unrestricted, CWT produces longer series of $W\left(s,\tau \right)$. In contrast, since DWT scales are based on powers of 2, the scale factor is dyadic in DWT^84^. More specifically, the value of s in the case of DWT (CWT) progresses in a geometric (arithmetic) order. As a result, even though DTW has faster processing capabilities and sufficient precision for time–frequency analysis, some scales will be missed^72^. According to Araghi et al.^84^, the DWT is better able to predict results for stationary time series with constant variance with respect to time than for nonstationary time series with seasonality components.

The main idea of the transform is to split the time series into various time scales. According to Adamowski et al.^85^, the wavelet transform of a time series is shown by:

2 $$W\left( {s,\tau } \right) = \frac{1}{\sqrt s }\mathop \smallint \limits_{ - \infty }^{ + \infty } x\left( t \right)\varphi^{*} \left( {\frac{t - \tau }{s}} \right)dt$$

3 $$\varphi_{{\left( {s,\tau } \right)}} \left( t \right) = \frac{1}{\sqrt s }\varphi \left( {\frac{t - \tau }{s}} \right)$$

where $W\left( {s,\tau } \right)$ denotes wavelet transform with $s,\tau$ defined as time shift and scale expansion respectively. Wavelet function and complex conjugate are denoted by $\varphi$ and $\varphi^{*}$ respectively. The mother wavelet is indicated by $\tau$ = 0 and $s$ = 1. Changes in scale, which enable the wavelet transform to capture both long and short frequency in the time series, are the cause of the wavelet transform's adaptability. The wavelet function $\left( \varphi \right)$ is localized in time and frequency with zero mean^72^. The Morlet wavelet, which is illustrated in Eq. (4), is one of the frequently employed complex wavelet functions in hydro-climatology^86^.

4 $$\varphi_{0} \left( \theta \right) = \pi^{ - 1/4} e^{{ - i\omega_{0} \theta }} e^{{ - \theta^{2} /2}}$$

where $\varphi_{0}$ represents Morlet wavelet, $\omega_{0}$ represents dimensionless frequency that controls resolutions of time and scale (higher (lower) ${\omega }_{0}$ scale resolution enhance (reduces) and time resolution reduces (enchance)). $\theta$ represents dimensionless time. It is worth mentioning that, for long (short) term oscillations in the time series, lower (higher) resolution is a practical option^72^. According to Araghi et al. and Grinsted et al.^72,87^ the Morlet wavelet's constrained scale helps it achieve great resolution in frequency. It also enables any time series' phase and amplitude to be separated.

#### Wavelet coherence approach

Cross wavelet transform's level of coherence in time–frequency space is assessed using the wavelet coherence approach^75^. In other words, the wavelet coherence approach first computes the correlation and then assess the connections between the pair of time series inside the time–frequency domain^34^. The wavelet coherence between time series x and y is described by Torrence and Webster^88^ as

5 $$R^{2} \left( {s,\tau } \right) = \frac{{\left| {S\left( {s^{ - 1} W_{xy} \left( {s,\tau } \right)} \right)} \right|^{2} }}{{S\left( {s^{ - 1} \left| {W_{x} \left( {s,\tau } \right)} \right|^{2} } \right) \times S\left( {s^{ - 1} \left| {W_{y} \left( {s,\tau } \right)} \right|^{2} } \right)}}$$

where $R^{2} \left( {s,\tau } \right)$ denotes the coherence coefficient and its value ranges between 0 (no coherence) and 1 (perfect coherence). Furthermore, the equation is very similar to the conventional correlation coefficient. $s$ denotes the scale expansion parameter and $\tau$ define the time-shift parameter (dimensionless). The cross wavelet transform of two time series is denoted by $W_{xy} \left( {s,\tau } \right)$, and S stands for smoothing operator, which is described as:

6 $$S\left( W \right) = S_{scale} (S_{time} \left( {W\left( {s,\tau } \right)} \right))$$

where $S_{scale}$ and $S_{time}$ stands for smoothing along the wavelet scale axis and time. The following equation, according to Torrence and Compo^89^ can be used to determine the best smoothing operator for the Morlet wavelet:

7 $$\begin{gathered} \left. {S_{time} \left( W \right)} \right|_{s} = \left. {\left( {W\left( {s,\tau } \right) \times c_{1}^{{\frac{{ - t^{2} }}{{2s^{2} }}}} } \right)} \right| _{s} , \hfill \\ \left. {S_{time} \left( W \right)} \right|_{\tau } = \left. {\left( {W\left( {s,\tau } \right) \times c_{2} \prod (0.6s} \right)} \right| _{\tau } \hfill \\ \end{gathered}$$

where the rectangle function is shown by $\pi$ and normalized constants are represented by $c_{1}$ and $c_{2}$. These two normalized constants are numerically calculated in practise since both convolutions are independently processed. The scale decorrelation length of 0.6 for the Morlet wavelet is calculated empirically^89^.

#### Global coherence approach

Partal and Küçük^90^ defined the time-averaged wavelet coherence coefficients as the global wavelet coherence (GWC) coefficient at scale’s as follows:

8 $$\overline{R}^{2} \left( s \right) = \frac{1}{n}\mathop \sum \limits_{\tau = 1}^{n} R^{2} \left( {s,\tau } \right)$$

where $n$ is total points within each time scale. By ignoring the impact of time, the global coherence coefficient can be used to assess the correlation between two time-series at various scales. According to Labat^89,91^, this parameter is helpful for assessing periodicity characteristics.

### Methodology

**Figure 3:**
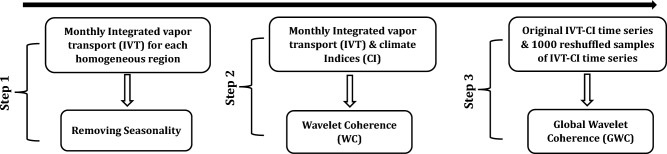
Schematic representation of the steps involved to conduct study.

**Figure 4:**
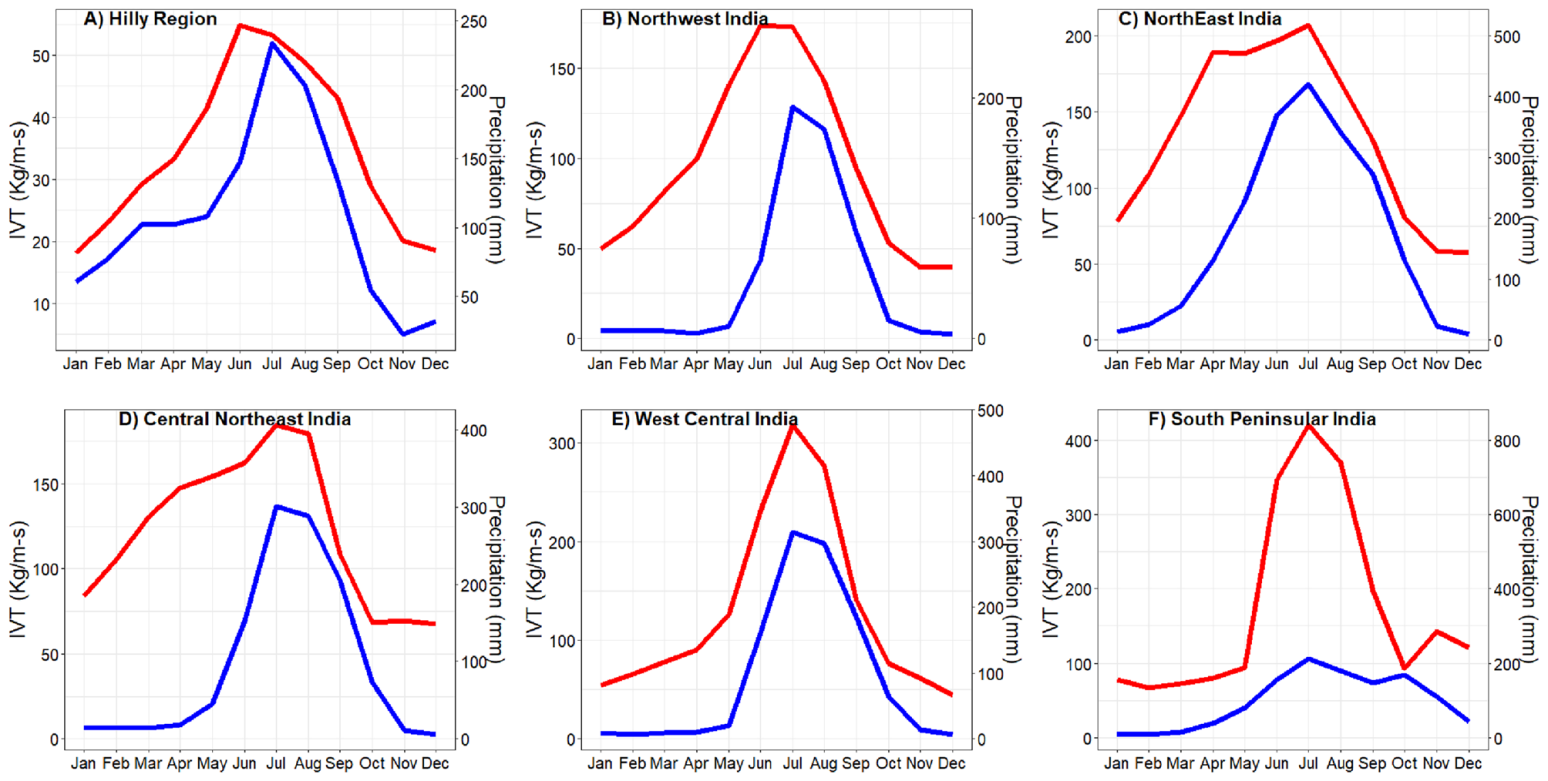
Monthly distribution of IVT (red color) and precipitation (blue color) across different homogeneous regions during 1951–2022.

Figure 3 illustrates the overall methodology used for the investigation. First, the integrated water vapour transport (IVT) was computed using ERA5 datasets for 1951–2022. The 6-hourly IVT dataset was averaged to the monthly temporal scale at each grid point to ensure their comparison with monthly climate indices data. Further, to depict the regional monthly IVT, the monthly IVT data at grids located within each homogenous region were averaged. In addition, daily precipitation data obtained from IMD was also averaged to the monthly temporal scale to evaluate its seasonality with respect to IVT across different homogeneous regions. The monthly precipitation and IVT distribution for 1951–2022 across different homogeneous regions are presented in Fig. 4. It can be noted that across all the homogeneous regions monthly IVT follow similar seasonal pattern as monthly precipitation irrespective of the magnitudes. Further, the presence of seasonality in monthly IVT (Fig. 4) is removed in order to avoid any misinterpretation while understanding its linkages with climate indices at a seasonal or smaller scale.

Secondly for each meteorologically homogeneous location, wavelet coherence (WC) and global wavelet coherence (GWC) between monthly IVT and climatic indices were assessed. Wavelet analysis was done in R Statistical Software using the biwavelet package^92^. Using 1000 ensemble surrogate pairs and lag-1 autoregressive coefficients as input datasets, the Monte Carlo approach is utilized to evaluate the statistical significance of wavelet coherence. The importance of the edge effects is then shown by calculating the significance level of each scale using values outside the influence cone^89^. Grinsted et al.^87^ also discovered that the significance level is strongly impacted by the resolution choice when computing the scale smoothing. With the least amount of processing, the scales per octave must be high enough to represent the rectangular shape of the scale smoothing operator. We chose 12 scales per octave for this investigation based on prior research^34,75, 89^. Furthermore, coherence is examined in the current study at a significance level of 5% and a confidence interval of > 95%.

Finally, a surrogate test is adopted to determine the statistical significance of the GWC values, as suggested by Agarwal et al.^93^. The time series (monthly IVT and teleconnection time series) are shuffled 1000 times at random while the distribution is left unchanged. This approach eliminates the possibility of any potential association between the time series by making them independent random series. The GWC values for each time series pair (monthly IVT and teleconnection) are then calculated for various periods. An empirical test distribution is used to compare the 1000 GWC values for the rearranged time series at each timescale to the GWC values of the original time series. Additionally, we determined that coherence cannot be due to chance if the GWC value at a specific timescale of the original time series is greater than the 95th percentile of the test distribution, using a 5% significance threshold.

## Results and discussion

### Linkages between AO & NAO and IVT

**Figure 5:**
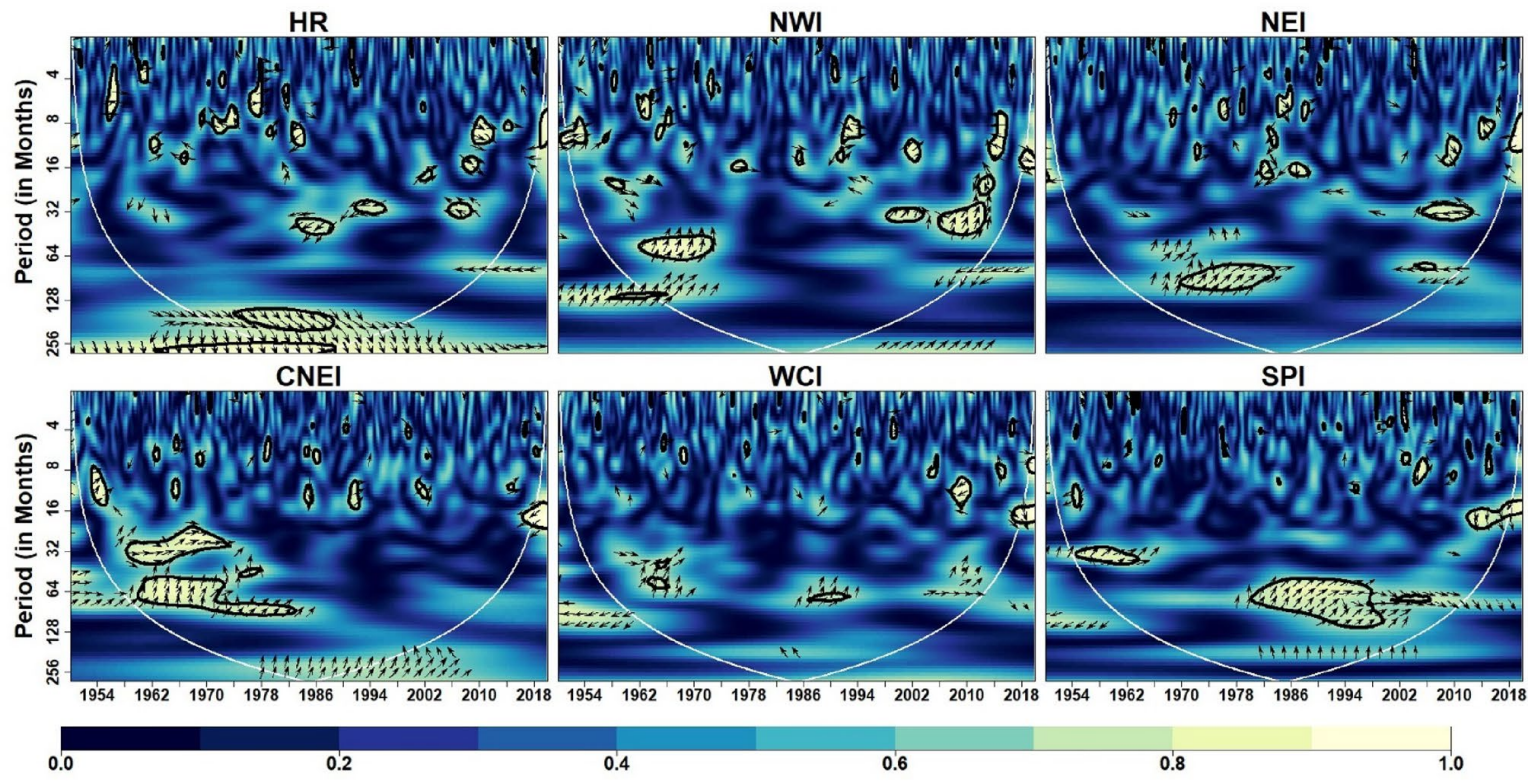
Wavelet Coherence between AO and IVT across different homogeneous regions. The thick contour indicates significant level. Color bar indicates coherence coefficient values. The black contour line denotes wavelet coherence at 95% significance level. The relative phase relationship between pair of series is shown by arrows. Left (right) pointing arrows indicate in-phase (anti-phase) relationship respectively.

Figure 5 depicts the coherence coefficient between AO and IVT in the homogeneous regions. At intra-annual scale (< 12 months), the coherence is reduced over with respect to time in HR, NWI, NEI and CNEI. At inter-annual scale (> 12 months but < 120 months), coherence is reduced over CNEI region whereas increased over SPI region with time. In addition, significant inter-decadal variability (> 120 months) is noticed over the HR region. Further, it can be observed that the WCI region exhibits less coherence across scales over time, indicating less influence of AO on monthly IVT over the WCI region. Similarly, global coherence (Fig. 13) suggests a significant AO-IVT linkage in NWI region at 8–16 months timescale while in SPI region at 64–96 months time-scale.

**Figure 6:**
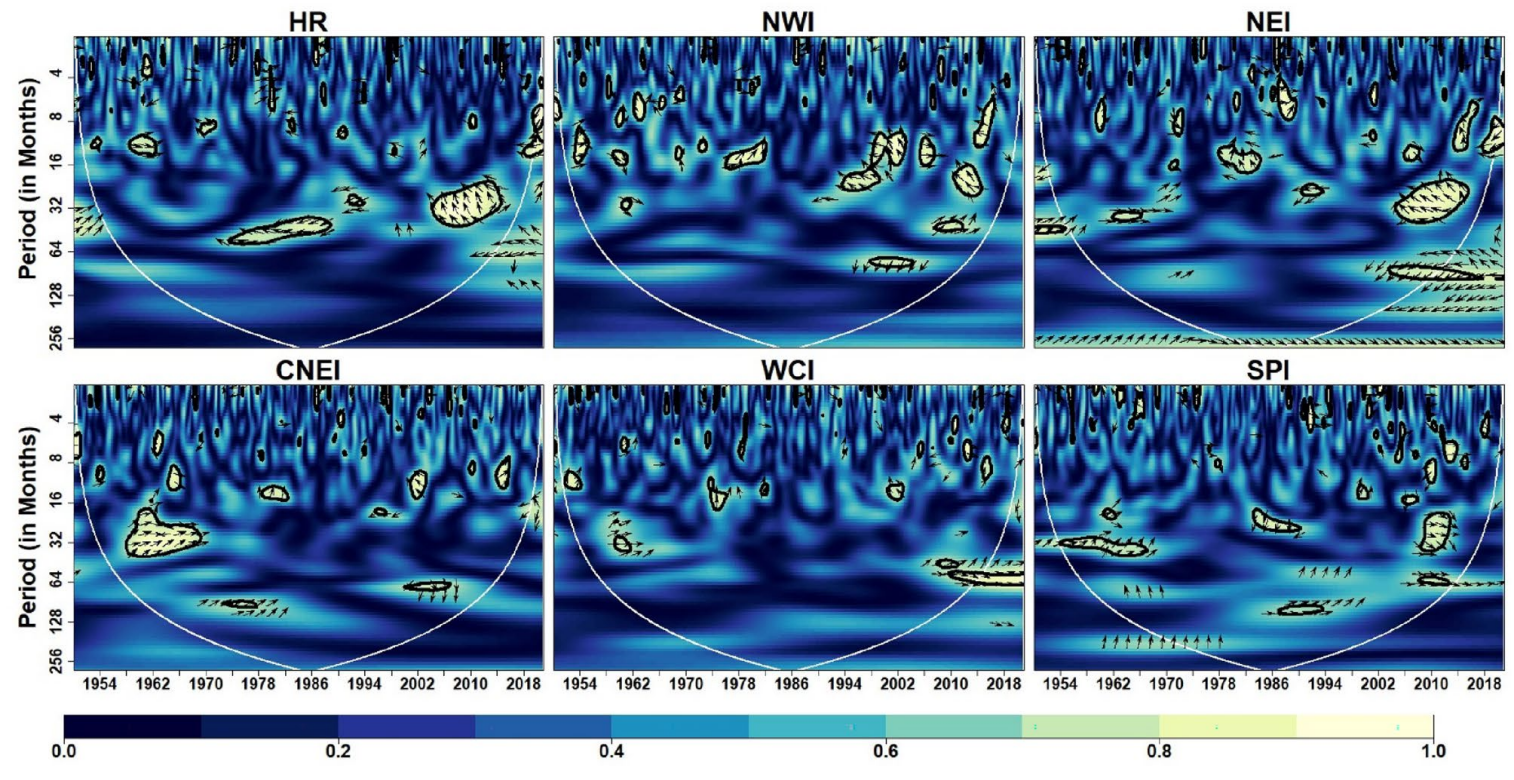
Wavelet coherence between NAO and IVT across different homogeneous regions. Same as Fig. 5.

Figure 6 depicts the coherence coefficient between NAO and monthly IVT in the homogeneous regions. At inter-annual scales, NAO affects monthly IVT across all regions. Similar to AO, NAO also has less influence on monthly IVT over WCI region. A strong coherence at time-scales of 16–64 months can be noted from 2000 onwards in HR, NWI, NEI and SPI regions. Global coherence depicts (Fig. 13) significant NAO-IVT linkages in HR (at timescale of 32–64 months), NWI (at timescales of 12–16 months) and SPI (at timescale of 3–4 months) regions.

Over the mid-high latitudes of the northern hemisphere, AO is one of the primary mode of atmospheric variability. Although it is always present, the boreal winter is when it is most active^94,95^. Over midlatitudes and polar regions, variations in sea level pressure are related to the several phases of AO. On the other hand, NAO is another significant mode of variability during winter in the Northern Hemisphere, which has a large impact on parts of Asia, Europe, and North America^96^. Walker and Bliss^97^ determined that the NAO is in charge of over 36% of the fluctuation in mean sea level pressure throughout the winter. Moreover, NAO and AO are intimately associated to one another (referred as AO/NAO) and demonstrate substantial connection^95^ during boreal winters thus influencing seasonal variability throughout the Northern Hemisphere^98^. According to numerous studies^98–100^ during the AO/NAO positive phase, the Asian westerly jet stream over the Middle East intensifies, causing intense moisture transport (IVT) from the Mediterranean, Caspian, and Arabian Sea towards the Indian subcontinent and winter precipitation over the northern part of India. In this study, both WC and GWC demonstrates that AO/NAO significantly influence IVT in NWI region of Indian subcontinent at inter-annual timescales. Our results are in line with studies that investigated the impact of AO/NAO on precipitation over NWI region^34,98–100^ and reported significant influence at inter-annual timescales. Over Hilly regions (HR), we found that IVT is significantly influenced by NAO at inter-annual scale. This can be explained by positive phase of NAO influencing Western Disturbance behaviour on interannual timescales causing intensification of subtropical jets and rise in moisture flux (by 40%) leading to increase winter precipitation (by 45%) over the Himalayas^101^. In addition, we found AO influencing IVT at inter-annual scale in SPI region. A recent study by Nagaraj and Srivastav^102^ also found AO influencing precipitation variability across SPI region. However, our study is the first to observe the linkages between AO and IVT across SPI region at inter-annual scale.

### Linkages between AMO and IVT

**Figure 7:**
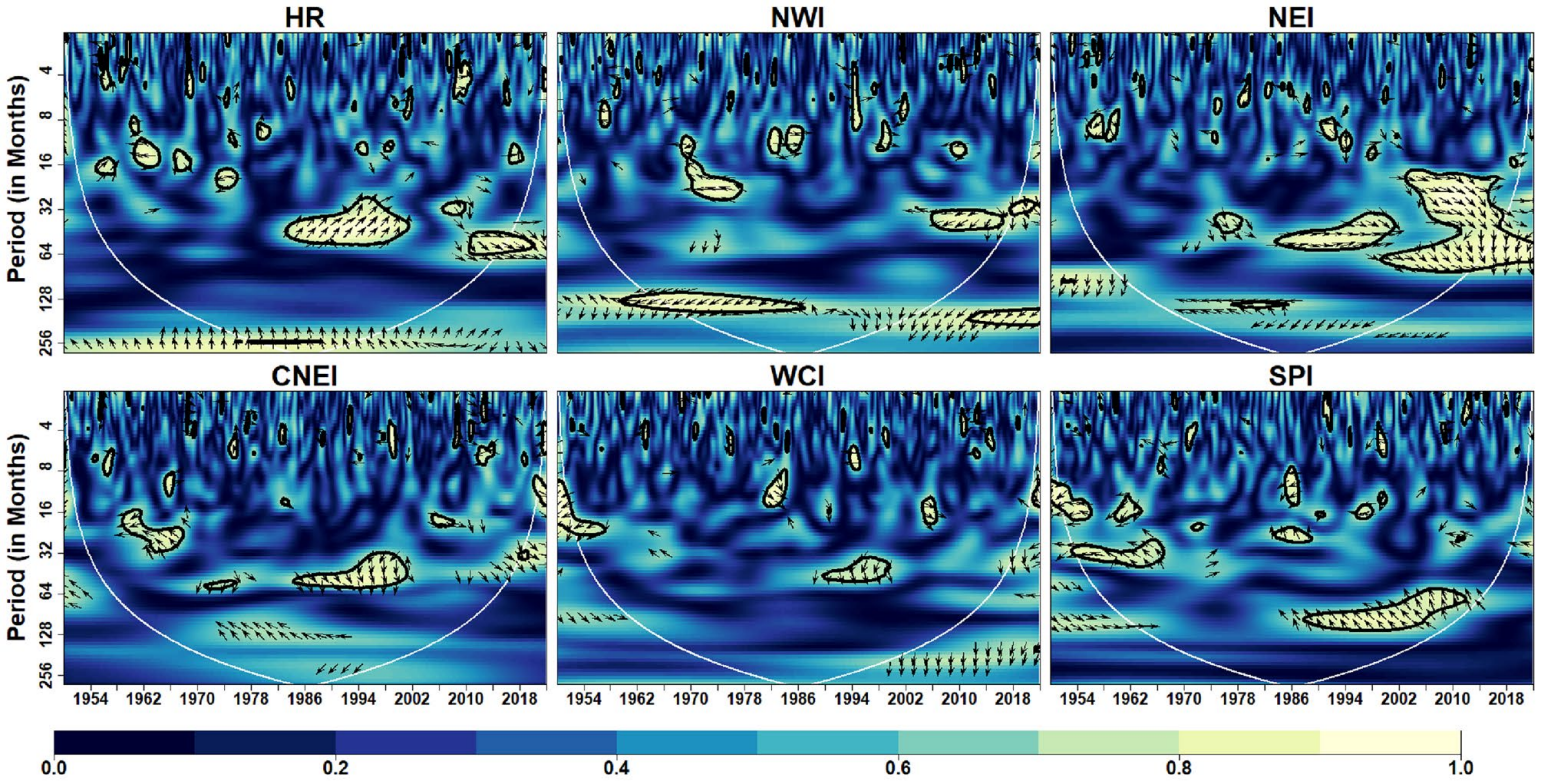
Wavelet coherence between AMO and IVT across different homogeneous regions. Same as Fig. 5.

Figure 7 depicts the wavelet coherence between AMO and IVT in the homogeneous regions. Similar to AO and NAO, AMO also has less influence on monthly IVT over WCI region. The coherence at inter-annual scale (> 12 but < 120 months) is increased significantly over the HR and NEI regions whereas reduced over CNEI region in recent decades. The prominent regions of high coherence at inter-decadal scale (> 120 months) are noticed over the NWI region. In addition, considerable inter-annual to decadal-scale coherence (64–128 months) is seen over the SPI region. AMO-IVT linkages as depicted from global coherence analysis (Fig. 13) at intra-annual time-scale (4–12 months) is observed only in HR and NEI regions, whereas significant AMO-IVT linkage at inter-annual scale (16–64 months) is observed in HR, NEI, CNEI and SPI regions.

It is generally recognised that the AMO, the dominant internal mode of Atlantic variability, is a significant contributor to Indian Summer Monsoon (ISM) variability on a multidecadal timescale^36,39, 103–106^. In addition, an AMO warm (cold) phase can cause the inter-tropical convergence zone (ITCZ) to move northward or southward over the ISM domain^106,107^, which can then strengthen or weaken the moisture transport toward the Indian subcontinent during the ISM. Our findings demonstrate significant AMO-IVT linkages at decadal scale in NWI region thus confirming Krishnamurthy and Krishnamurthy’s^104^ claim that there is a link between AMO and Indian monsoon rainfall at the decadal scale. Over NEI, CNEI and SPI regions our results suggest AMO-IVT linkages at intra- to inter-annual timescales. It is worth mentioning that monsoons in India are related to tropical easterly and subtropical westerly jet streams. Circum-global teleconnection (CGT), also known as zonal teleconnection pattern, has been used in studies to examine the substantial interannual variability in subtropical jet streams in the past^108,109^. According to Ding and Wang, and Saeed et al.^110,111^, Atlantic-ISM interaction at intra- and inter-annual timescales is through the atlantic modulation of CGT and circulation responses across west central Asia.

### Linkages between PDO and IVT

**Figure 8:**
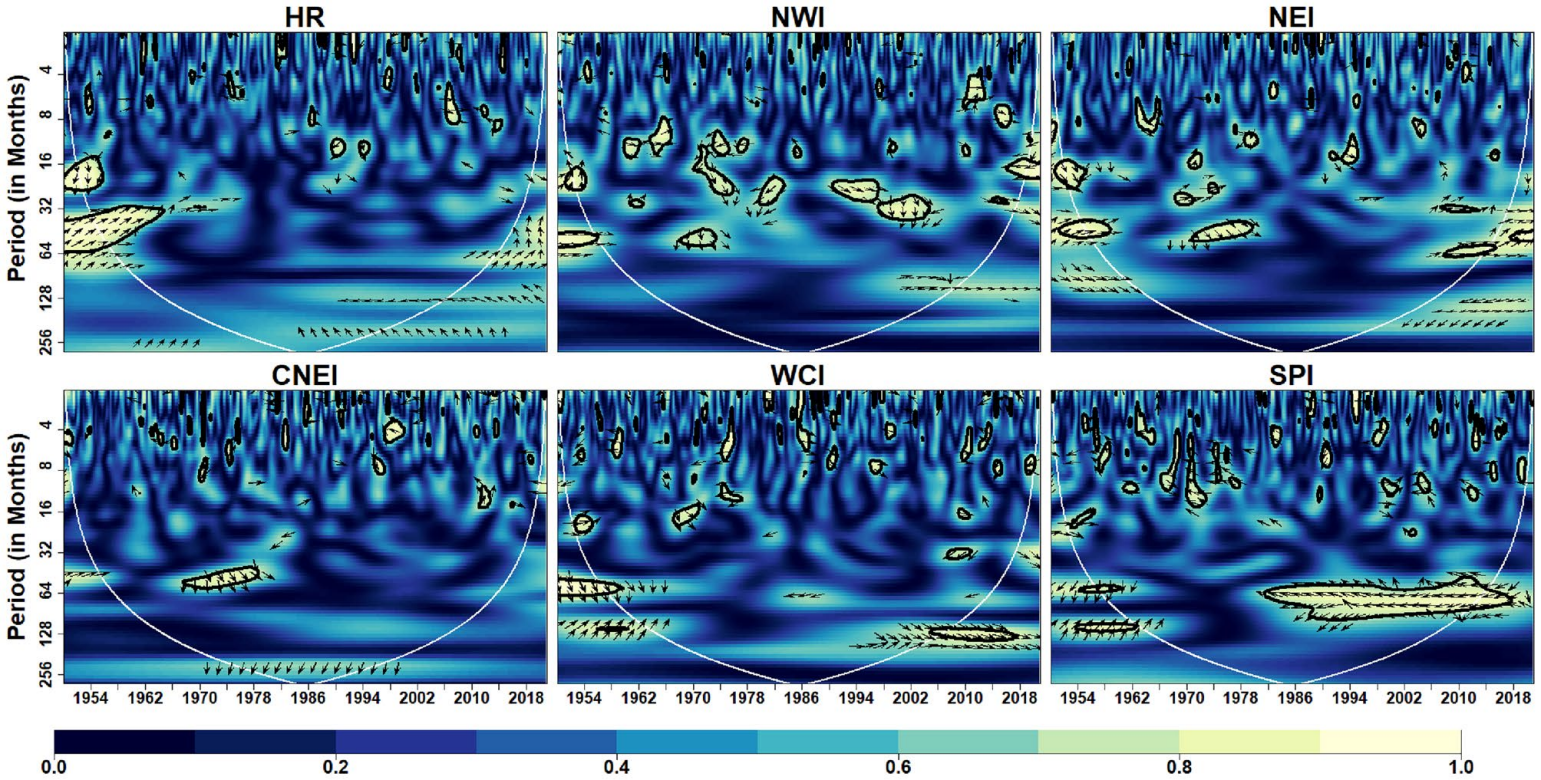
Wavelet coherence between IVT and PDO across different homogeneous regions. Same as Fig. 5.

Figure 8 depicts the wavelet coherence between PDO and IVT in the homogeneous regions. Analysis shows that PDO is significantly influenced at inter-annual scale (16–64 months) across all regions at different durations. Other scales are intermittently observed over the WCI and SPI regions over different years, although substantial coherence is observed at inter-annual to inter-decadal time scales (64–128 months) in the SPI region from 1980 onwards. In addition, it can be clearly seen that except SPI no other region exhibits continuing coherence between monthly IVT and PDO on any time scale. Further, global wavelet analysis suggest significant PDO-IVT linkages exhibit in NWI (at timescale of 12–32 months), NEI (at timescales of 16–64 months), CNEI (at timescales of < 8 months) and SPI regions (at timescale of 3–5, 8–16 and 60–70 months).

One of the important phenomena in the Northern Hemisphere, the PDO is known for the heat differential in the North Pacific Ocean and has a periodicity on the order of decadal scale^42^. The Walker circulation changes over the Indian and Pacific Oceans depending on the PDO phase. The Indian summer monsoon rainfall (ISMR) deficit (surplus) is associated with the positive (negative) phases of the PDO, which also strengthen (suppress) the association between the ISMR and ENSO, according to Krishnamurthy and Krishnamurthy and, Krishnan and Sugi^42,112^. In addition, PDO under the influence of El Nino, influence Indian precipitation at inter-annual scale. Our findings also indicates significant coherence between PDO and atmospheric moisture transport at inter-annual scale over whole India regions except HR and WCI. However, over SPI region our results suggests that PDO significantly influence atmospheric moisture transport at decadal scale. Thus, confirming the assertion made by Krishnamurthy and Krishnamurthy^104^ who found warm phase of pure decadal component of PDO resisting atmospheric moisture to transport beyond 20° N over the Indian monsoon region by meridional winds coming from the North Pacific. In addition, our study is the first to observe the linkages between PDO and monthly IVT in CNEI and SPI regions at intra-annual scale.

### Linkages between IOD and IVT

**Figure 9:**
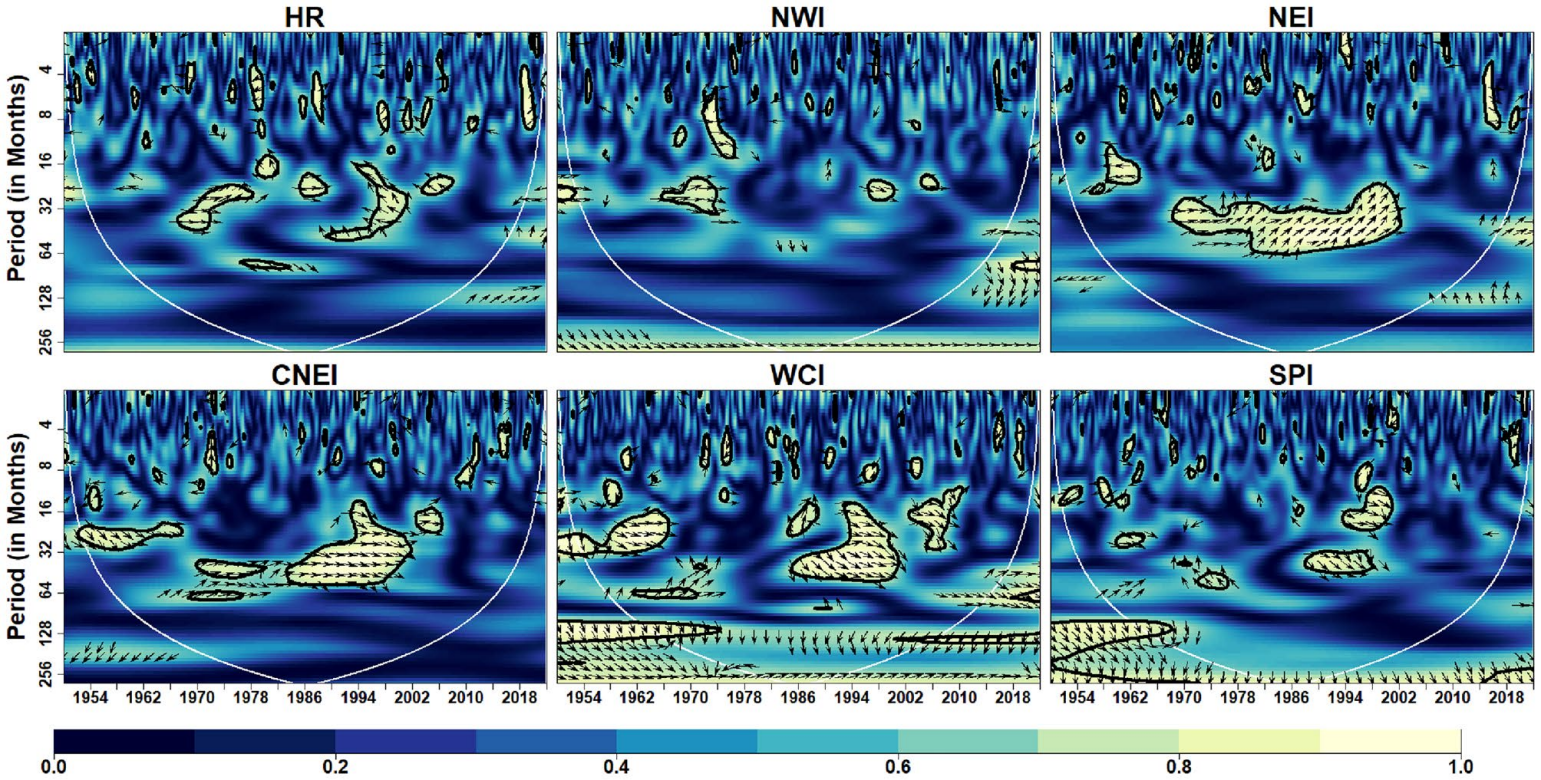
Wavelet coherence between IVT and IOD across different homogeneous regions. Same as Fig. 5.

Figure 9 depicts the wavelet coherence between IOD and monthly IVT in the homogeneous regions. The impact of IOD on IVT for whole India is noticeable within 8–16 months timescale and 16–64 months timescale (intra- and inter-annual variability) during different years. Over CNEI, HR and NEI regions, a strong inter-annual coherence (32–64 months) are observed during 1970–2000 period.

In addition, it can be observed that the WCI region exhibits strong coherence across scales over time, indicating strong influence of IOD on the monthly IVT over the WCI region. Among all homogeneous regions, it can be noticed that NWI region experience less influence of IOD on monthly IVT across different timescales. Similarly, global coherence suggests IOD-IVT significant linkage exist mostly at intra- and inter-annual time-scale for all region except SPI (Fig. 13). In WCI region significant IOD-IVT linkage also exhibit at inter-decadal scale (> 120 months).

IOD is a phenomenon that exhibit inter-annual variability^113^ and is characterised by opposite western and eastern Indian Ocean surface temperature anomaly^114^. It is important to note that the positive phase of the IOD amplifies the precipitation over India by increasing moisture transport from the southeast Indian Ocean and causing high convergence over the Bay of Bengal^115,116^. Additionally, Cherchi et al.^117^ emphasized the significance of moisture transport from the western polar area to the Indian subcontinent during the IOD's positive phase. Studies in past have indicated significant influence of IOD on the precipitation over India by modulating meridional monsoon circulation^118^. For instance, Ashok et al.^48^ and Das et al.^34^ indicated influence of IOD on precipitation at inter-annual scale and intra-annual to inter annual scale respectively. The present study indicates that IOD has a considerable impact on moisture transport at the intra-annual to inter-annual scale across all regions. However, the WCI region has also seen effects on an inter-decadal scale. Several research in the past have investigated the cause of decadal fluctuation in IOD. For instance, Ashok^119^ attributed decadal IOD variability to ocean dynamics i.e., 20 °C isotherm depth anomaly whereas Krishnamurthy and Krishnamurthy^40^ attributed decadal IOD variability to PDO. On contrary, decadal variability in ENSO-IOD relation was found to be primary cause behind decadal variability in IOD by Song et al.^120^. Despite the progress, more study is still needed to fully comprehend how regional physical factors and the decadal variability of IOD interact.

### Linkages between ENSO and IVT

**Figure 10:**
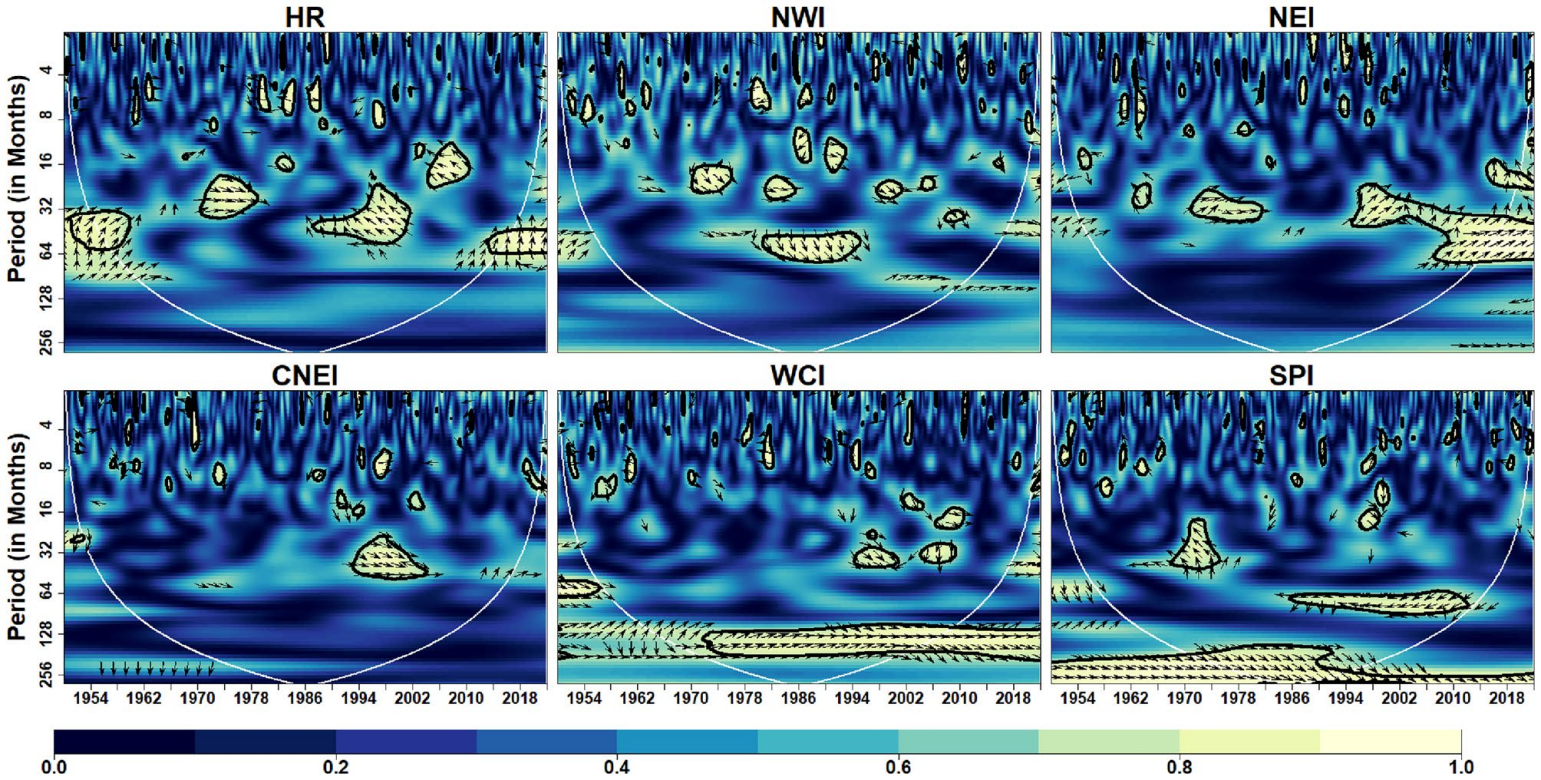
Wavelet coherence between NINO34 and IVT across different homogeneous regions. Same as Fig. 5.

**Figure 11:**
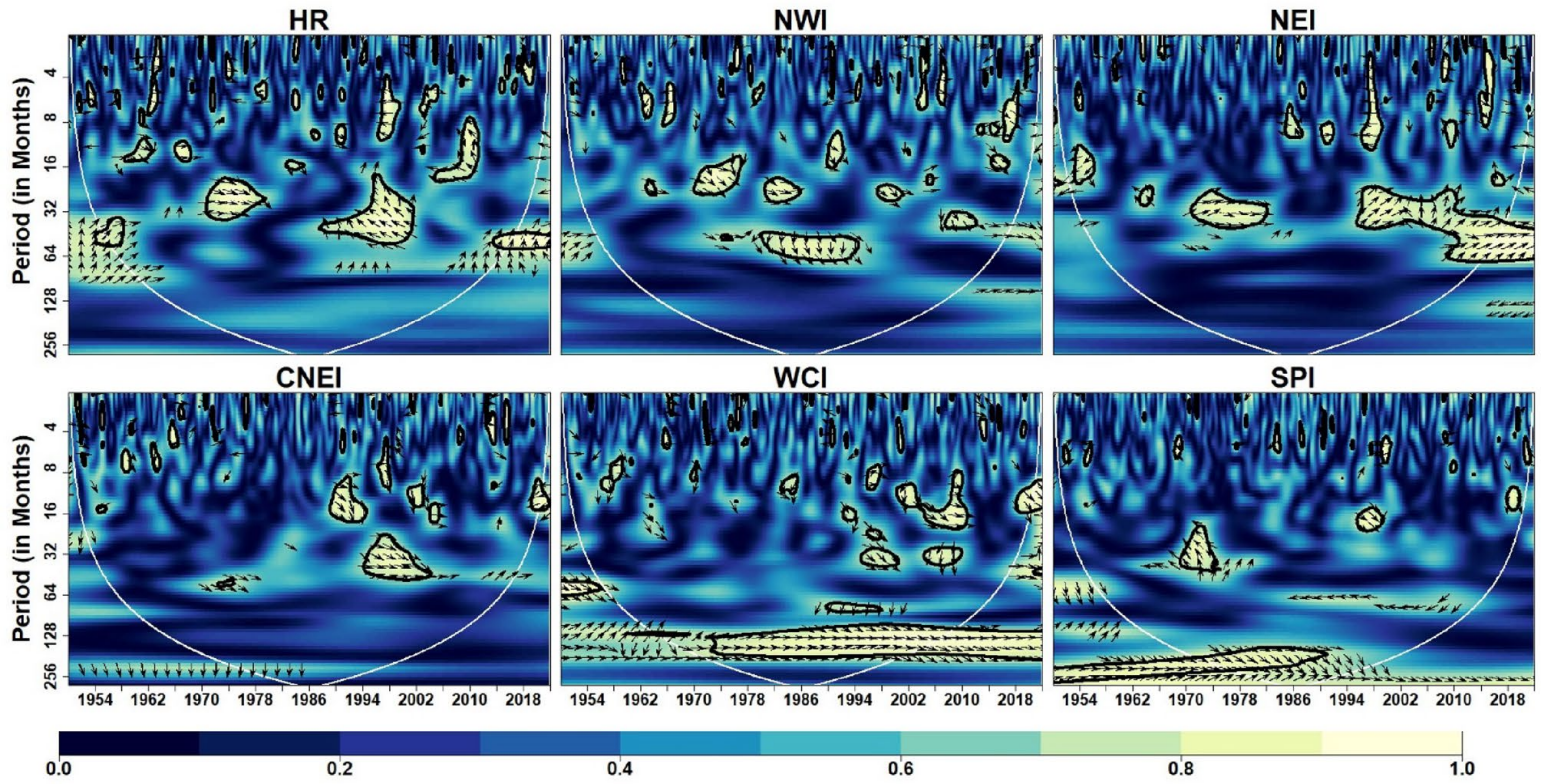
Wavelet coherence between MEI and IVT across different homogeneous regions. Same as Fig. 5.

**Figure 12:**
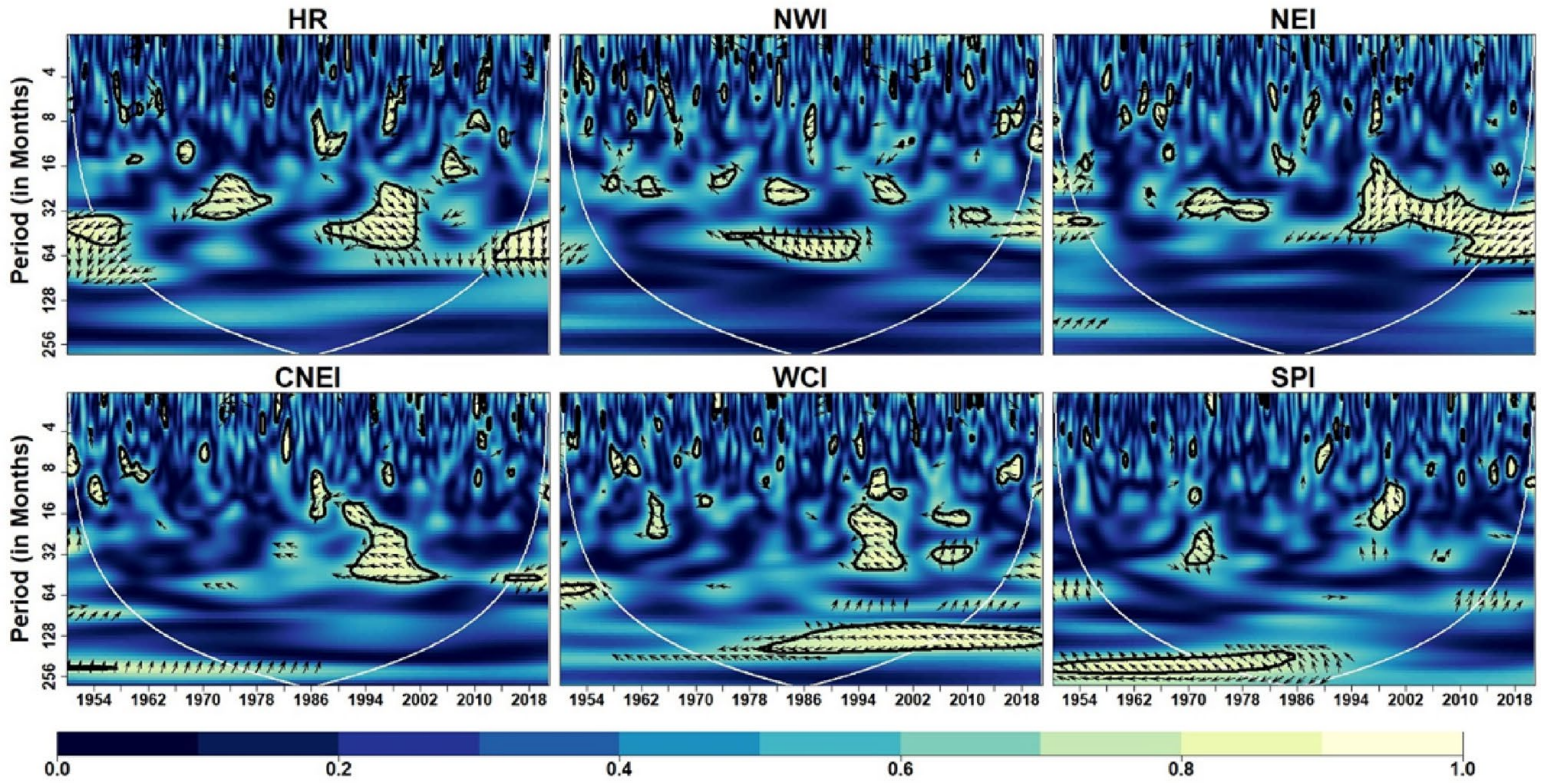
Wavelet coherence between SOI and IVT across different homogeneous regions. Same as Fig. 5.

**Figure 13:**
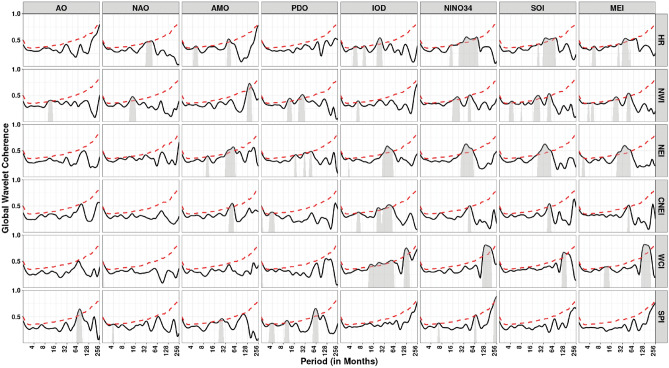
Global wavelet coherence between climate indices and IVT across different homogeneous regions. Solid black lines depicts GWC values. The 95th percentile value of the test distribution is shown as red dotted line and substantial coherence (95% significance level) at different time-scale are marked in grey.

We used NINO3.4, SOI, and MEI as three different ENSO indices to depict the ENSO cycle. Figure 10 depicts the coherence coefficient between NINO3.4 and IVT in the homogeneous regions. The impact of NINO3.4 (SST anomaly) on IVT for HR, NWI and NEI regions is noticeable at 16–64 months timescale over most of the time during 1951–2022. The substantial regions of strong coherence in case of MEI (Fig. 11) are similar to NINO3.4 at inter-decadal time-scale (> 128 months) in WCI and SPI regions. However, their exist small number of inter-annual variability in case of NINO3.4. Figure 11 depicts the coherence coefficient between MEI and IVT in the homogeneous regions. Similar to NINO3.4, MEI also exhibit significant coherence with IVT in HR, NWI and NEI regions at inter-annual scale (16–64 months) during different years. Moreover, MEI influence IVT in HR, NWI and NEI regions at scale < 16 months from 2000 onwards whereas a strong inter-decadal variability (> 128 months) is observed in WCI and SPI regions. Figure 12 depicts the coherence coefficient between SOI and IVT in the homogeneous regions. It is observed that the coherence structure found for MEI and NINO3.4 closely resembles to the coherence structure seen in all regions. Similarly, global coherence analysis (Fig. 13) suggests, significant MEI-IVT linkages in HR, NWI and NEI region at time-scale of < 8 months whereas at time-scale of 8–16 months only WCI region show significant linkages.

At time-scale ranging from 16 to 64 months HR, NWI, NEI and CNEI regions showed significant MEI-IVT linkages. In WCI region significant MEI-IVT linkage also exhibit at inter-decadal scale (> 120 months). Further, it is observed that the significant coherence structure between NINO3.4 and IVT, and SOI and IVT is similar to MEI and IVT in all regions except SPI region where significant NINO3.4-IVT linkages exhibit at all time-scales.

According to Chakraborty and Singhai^10^ one of the main climate variability modes, ENSO, accounts for around 29% of the ISM's interannual variability. In general, an El Nino/La Nina also referred as two phases of ENSO cycle are connected to decline/increase in ISMR respectively. The ENSO-induced large-scale fluctuations in surface pressure between the Pacific and the Indian Ocean have an impact on the moisture-laden winds and precipitation over India^10^. NINO3.4, SOI, and MEI are used to depict ENSO phenomena in the current study. Over all the regions, the ENSO has a substantial inter-annual influence. According to Ashok et al.^48,121^ the ENSO is the most important ocean–atmosphere event on inter-annual time scales in relation to moisture transport and precipitation variability over India. A recent study by Syed and Hannachi^122^ revealed dominant mode of moisture transport exhibiting inter-annual variability was linked to El Nino conditions in the Pacific Ocean. Our findings also revealed existence of significant influence at intra-annual scale between ENSO and moisture transport in HR and NWI regions. These results are in line with Neena et al.^123^ who investigated sub-seasonal modes of moisture transport and revealed that ENSO modulate mean IVT over monsoon domain by modulating the intra-seasonal modes of IVT and convection. Further, we also observed significant coherence structure at decadal scale in WCI and SPI regions. The reason behind decadal variability has been addressed by several studies in past. For instance, Goswami^35^ attributed interdecadal variability of Indian monsoons to interdecadal variability of ENSO. Moreover, a study by Meinke et al.^124^ noted that ENSO signals had an effect on precipitation variability on both decadal and interdecadal time scales.

## Conclusions

**Figure 14:**
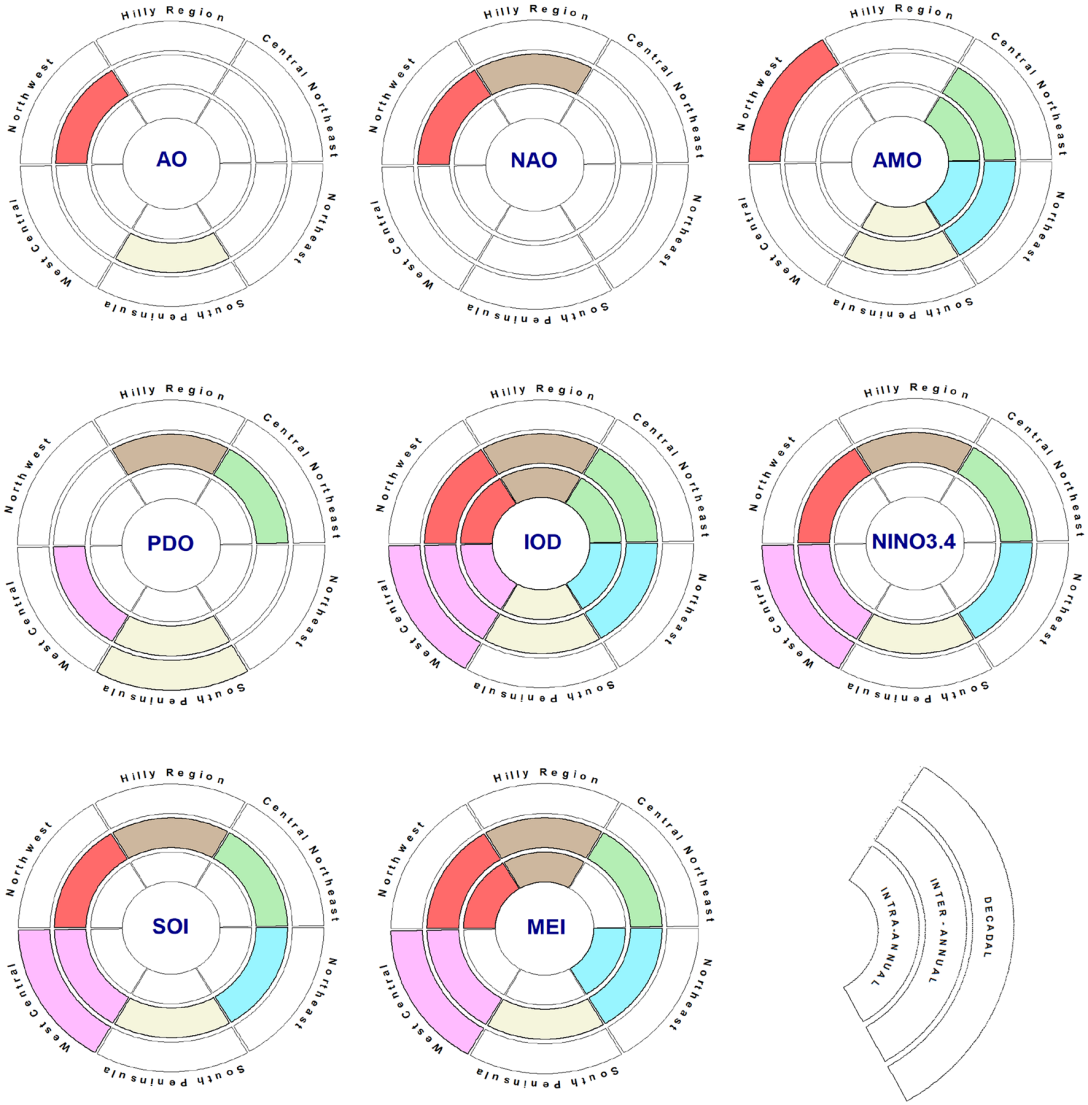
Indian atmospheric moisture transport teleconnections at various timescales across homogeneous regions. The colors match the homogeneous zones depicted in Fig. 1. Regardless of the coherence magnitude, the presence of color in the region segment suggests a strong connection between teleconnection and atmospheric moisture transport over region. Each individual circle segment depicts the temporal scale.

The current study unravels the linkages between climate teleconnection and atmospheric moisture transport over homogeneous regions in Indian subcontinent. In order to unravel how eight large scale climate oscillations (AO, NAO, AMO, PDO, IOD, NINO3.4, SOI, MEI) are linked to atmospheric moisture transport at different timescales, this study incorporated wavelet techniques. For various timescales, our study found geographical diversity in linkages across Indian subcontinent (Fig. 14). At the intra-annual scale, the IOD emerges as a key determinant of atmospheric moisture transport across the entirety of India. The IOD's significant influence highlights its importance in shaping seasonal patterns of atmospheric moisture transport. Furthermore, MEI has a significant impact on atmospheric moisture transport, particularly in the hilly region, Northwest India, and Northeast India. During the intra-annual timeframe, the AMO has a significant influence on atmospheric moisture transport in Northeast India, Central Northeast India, and the Southern Peninsula region.

At the interannual scale, both ENSO and IOD become prominent, influencing atmospheric moisture transport across all regions of India. The PDO comes into play, influencing moisture transport in various regions, with the exception of the hilly region and West Central India. Notably, the AMO's influence becomes more pronounced during this timeframe, significantly affecting moisture transport in Northeast India and the Southern Peninsula. At the interannual scale, the AO emerges as a significant factor influencing moisture transport in specific regions of India. Its primary impact is felt in the country's Northwest and South peninsula region. At the same timescale, the NAO has an impact on moisture transport in different regions. The NAO's impact is particularly noticeable in India's northwest and hilly regions. The AMO retains its influence on the interdecadal scale, particularly in Northwest India. Meanwhile, the PDO takes center stage, influencing moisture transport patterns in the Southern Peninsula region over long decadal time scales. It is worth noting that both IOD and ENSO influence persist on an inter-decadal timescale over the Western Central India region.

Overall, our study highlights the influence of climate oscillations on atmospheric moisture transport at various timescales over different homogeneous regions of India. The findings of this study will offer sufficient context and insight to aid in predicting upcoming atmospheric moisture transport and associated precipitation under the impact of large-scale climatic oscillations. The current study, however, does not discuss the specific physical mechanisms involved in atmospheric moisture transport variability influenced by climate oscillations at different timescales, but recommends it for future research.

## Data Availability

All data used for this study are freely available. Precipitation data from IMD are available from https://www.imdpune.gov.in/cmpg/Griddata/Rainfall_25_NetCDF.html ERA5 reanalysis data are obtained from the website: https://cds.climate.copernicus.eu/#!/search?text=ERA5&type=dataset. Climate indices dataset is accessible from https://www.ncei.noaa.gov/access/monitoring/nao/.
